# Acute hypoxia influences collagen and matrix metalloproteinase expression by human keratoconus cells *in vitro*

**DOI:** 10.1371/journal.pone.0176017

**Published:** 2017-04-20

**Authors:** Tina B. McKay, Jesper Hjortdal, Shrestha Priyadarsini, Dimitrios Karamichos

**Affiliations:** 1 Department of Cell Biology, University of Oklahoma Health Sciences Center, Oklahoma City, Oklahoma, United States of America; 2 Department of Ophthalmology, Aarhus University Hospital, Aarhus, Denmark; 3 Department of Ophthalmology/Dean McGee Eye Institute, University of Oklahoma Health Sciences Center, Oklahoma City, Oklahoma, United States of America; Cedars-Sinai Medical Center, UNITED STATES

## Abstract

Keratoconus (KC) is a progressive corneal ectasia linked to thinning of the central cornea. Hard contact lenses, rigid gas permeable lenses, and scleral lenses are the primary treatment modalities for early to mid- stages of KC to correct refractive error and astigmatism that develops as a result of an irregular corneal structure. These treatments are associated with significant drawbacks, including reduced availability of the tear film and oxygen to the corneal epithelium and stroma. However, it remains unknown whether hypoxia affects corneal integrity in the KC pathobiology. A number of studies have associated elevated oxidative stress with KC both *in vitro* and *ex vivo*. We hypothesized that KC-derived corneal fibroblasts are more susceptible to hypoxia-induced oxidative stress compared to healthy controls leading to exacerbation of corneal thinning in KC. This study investigated the effects of hypoxia on ECM secretion, assembly, and matrix metalloproteinase (MMP) expression in human corneal fibroblasts from healthy controls (HCFs) and KC patients (HKCs) *in vitro*. HCFs and HKCs were cultured in 3D constructs for 3 weeks and maintained or transferred to normoxic (21% O_2_) or hypoxic (2% O_2_) conditions, respectively, for 1 additional week. At the 4 week time-point, constructs were isolated and probed for Collagen I, III, and V, keratocan and MMP-1, -2, -3, -9, and -13, as well as hypoxia markers, hypoxia inducible factor-1α and lactoferrin. Conditioned media was also collected and probed for Collagen I, III, and V by Western blot. Thickness of the ECM assembled by HCFs and HKCs was measured using immunofluorescence microscopy. Results showed that hypoxia significantly reduced Collagen I secretion in HKCs, as well as upregulated the expression of MMP-1 and -2 with no significant effects on MMP-3, -9, or -13. ECM thickness was reduced in both cell types following 1 week in a low oxygen environment. Our study shows that hypoxia influences collagen and MMP expression by HKCs, which may have consequential effects on ECM structure in the context of KC.

## Introduction

Maintaining a transparent, well-organized cornea of sustainable thickness is essential for quality vision. Resident keratocytes synthesize, deposit, and remodel the ECM within the corneal stroma, which is important in providing the structural rigidity and optical properties required for proper tissue function. In KC, the central corneal thickness is reduced 8–20% with averages measured at 0.52 +/- 0.04 mm compared to a normal thickness of 0.56 +/- 0.02 mm [1]. Even slight thinning of the corneal stroma can lead to a bulging phenomenon that distorts transmission of light to the retina leading to defects in visual acuity. KC is often characterized by the formation of a cone-like cornea at the region with increased thinning near the central apex in the presence of normal intraocular pressure. Up to 78% of KC patients are prescribed treatment modalities that reduce oxygen levels in the stroma, such as hard contact lenses, rigid gas permeable lenses, or scleral lenses, at some point during progression of the disease in order to correct for changes in visual acuity [2–4].

It was first posited in 1968 by Hartstein, et al. that contact lens use may be an environmental risk for the development of KC [5]. Since then, studies have supported a role for the promotion of KC development by hard contact lenses in a subpopulation of individuals [6, 7]. Several studies have shown that both KC patients and contact lens wearers have significantly reduced corneal thickness compared to healthy controls and non-contact lens wearers [8–10]. Contact lenses are known to reduce the amount of oxygen that reaches the cornea leading to corneal edema [11, 12]. The effects of hypoxia on ECM assembly have yet to be explored at the molecular level in the KC pathobiology. In our previous demographic study, we identified a sub-population of KC patients with increased maximum corneal curvature (Kmax) of 95 diopters within contact lens wearers compared to 65 and 60 diopters in the group prescribed glasses or without treatment, respectively [13]. Increased curvature of the central cornea can impair proper contact lens fitting and accelerate the need for corneal keratoplasty [2, 8]. More severe cases require use of a specialized contact lens that fits to the sclera in order to correct for the irregular corneal structure. It remains unclear if contact lenses play any role in promoting KC development or progression.

Oxygen levels play an essential role in cellular metabolism and fluctuations are known to regulate the stability and expression of a number of proteins, including the hypoxia-inducible factor-1 (HIF-1) complex consisting of HIF-1α and aryl receptor nuclear transporter (ARNT) [14]. HIF-1α forms a heterodimer with ARNT, also known as HIF-1β, which as a complex translocates to the nucleus and activates genes associated with metabolism, angiogenic factors, and other transcription factors [15, 16]. Unlike ARNT whose constitutive expression and stabilization is fairly constant, HIF-1α is subjected to ubiquitination and degradation during normoxia and is only stabilized in the absence of oxygen [17].

Given that the cornea is an avascular tissue, it relies primarily on atmospheric oxygen and oxygen dissolved within the aqueous humor and tear film in order to maintain ATP production via oxidative phosphorylation [18]. We have previously shown that HKCs exhibit increased oxidative stress and favor aerobic glycolysis and lactate production with reduced citric acid cycle intermediates even at sufficient oxygen levels compared to HCFs [19]. These data suggest that HKCs are less reliant on oxidative phosphorylation for energy production. Hypoxia results in reduction of the terminal electron acceptor (O_2_) and therefore may increase the oxidative stress within a cell with the lack of sufficient terminal electron acceptors required during oxidative phosphorylation. A cell’s counter-mechanism to enable survival during hypoxia is to stabilize HIF-1α which then upregulates aerobic glycolysis to generate ATP for the continual energy demand [17, 20].

Our current study investigated the effects of local hypoxia on protein expression and signaling, matrix secretion, remodeling, and deposition by corneal fibroblasts from KC patients compared to healthy controls in an effort to determine if treatment modalities that reduce oxygen levels affect stromal thinning in KC. We also investigated the effects of hypoxia on malonyl CoA levels, an important inhibitor of lipid peroxidation, which is synthesized from acetyl CoA by acetyl CoA carboxylase (ACC) and degraded by malonyl CoA decarboxylase (MCD). These enzymes play an important role in regulating mitochondrial-mediated lipid peroxidation during pathological conditions with an inflammatory component, such as cancer and diabetes [21, 22]. Our findings revealed the role of hypoxia in regulating ECM structure and malonyl CoA levels in KC suggesting that the corneal stroma may be sensitive to the effects of hypoxia.

## Materials and methods

### Ethics and inclusion criteria

All experiments were completed with IRB approval (Protocol # 3450). This study met the tenets of the Declaration of Helsinki. Written permission was obtained prior to collection of corneas following corneal transplantation or death for HKCs or HCFs, respectively. None of the transplant donors were from a vulnerable population and all donors or next of kin provided written informed consent that was freely given. Tissue was collected from the period of 2012 to 2016. Cells were isolated from de-identified tissue samples with only the age and gender of the donor provided. Corneal tissue with no history of ocular disease was considered healthy. KC corneal tissue was provided post-corneal transplantation and excluded patients who had previously undergone collagen crosslinking.

### Isolation of primary corneal fibroblasts

Primary corneal fibroblasts were isolated as previously described [23, 24]. Briefly, corneal tissue from cadavers was provided by the National Disease Research Interchange (NDRI; Philadelphia, PA) with no identifiers linked to patient. Primary corneal fibroblasts were isolated by removing epithelial and endothelial layers with a sterile surgical scalpel. Tissue was cut into small ~2x2x2mm pieces and incubated in sterile flasks to promote adhesion. Explants were then supplemented with EMEM containing 10% fetal bovine serum (FBS, Atlanta biologicals, Flowery Branch, GA) and antibiotic/antimycotic (anti/anti, Life Technologies, Grand Island, NY) and allowed to incubate at 37°C/5% CO_2_ for 2–6 weeks until cells migrated from explant.

### 3D *in vitro* model

As previously described [19, 24], primary corneal fibroblasts from healthy controls (HCFs) and KC patients (HKCs) were seeded at 10^6^ cells/well in polycarbonate transwell plates containing 3mL total of EMEM (ATCC, Manassas, VA) (1.5mL top well and 1.5mL bottom well) with 10% FBS and antibiotic/antimycotic. At t = 24 hours, EMEM (10% FBS+anti/anti) was prepared with 0.5mM of 2-O-α-D-glucopyranosyl-L-ascorbic acid (American Custom Chemicals Corporation, San Diego, CA) and added to constructs following filter sterilization. Constructs were supplemented with fresh media 3X per week for 4 weeks total to promote native ECM assembly.

### Hypoxic environment

Constructs under hypoxic conditions were maintained in a hypoxic incubator (New Brunswick Galaxy 14S, Eppendorf, VWR International, Bataria, IL) at 2% O2/5% CO2/37°C for 1 week following 3 weeks at normoxic conditions (~21% O2/5% CO2/37°C) (S1 Fig). Media was changed every other day for 3 weeks and every day for the last week in normoxic and hypoxic conditions. Hypoxia-maintained cultures were exposed to <5minutes in normoxia.

### Media collection and cell lysis

Isolation of media was performed at the end of week 1 from constructs in normoxic (21%) and hypoxic (2%) oxygen levels. Cell lysis was performed at the end of week 4 (post-1 week in hypoxia) using 1X RIPA buffer containing protease inhibitors, as previously described [25]. Briefly, constructs were isolated and washed 1X with PBS. Constructs were removed gently by forceps and spatula and transferred to a clean microcentrifuge tube containing 1x RIPA and protease inhibitors and incubated on ice for 15 minutes followed by centrifugation (12000 rpm, 4°C, 15 minutes) to pellet cell debris. The supernatent was isolated and stored at -20°C until further use. Determination of the pH of the isolated media was performed by collecting media into sterile Eppendorf tubes and then measured using a calibrated pH/Ion 510 meter (Fisher). Media was changed daily during the last week to minimize pH-induced effects of hypoxia (S2 Fig).

### Protein quantification and western blot

A BCA Assay (ThermoScientific, Rockford, IL) was used to measure protein concentration prior to Western blotting. About 30μg of total protein was loaded onto a gradient gel (4%-20%) and electrophoresed at 140V for 1.5 hours and transferred onto a nitrocellulose membrane at 100V in 1 hour on ice. Membrane was blocked in 5% BSA or 5% dry milk for 1 hour at room temperature and probed with antibodies at 1:500–1000 ratio in 1% BSA solutions overnight at 4°C. The following antibodies were purchased from Abcam (ab, Cambridge, MA), Sigma Aldrich, or ThermoScientific (Thermo): Collagen I (ab34710), Collagen III (ab83829), Collagen V (ab7046), glyceraldehyde-3-phosphate dehydrogenase (GAPDH) (ab9485), MMP-1 (ab38929), MMP-2 (ab37150), MMP-3 (ab52915), MMP-8 (ab81286), MMP-9 (ab38898), MMP-13 (ab39012), HIF-1α (ab85886), aryl hydrocarbon receptor nuclear translocator (ARNT) (Sigma, HPA001759), keratocan (ab113115), β-actin (ab8227), pFAK (phospho Y397) (ab39967), FAK (ab40794), malonyl CoA decarboxylase (MCD) (Thermo, PA5-22081), and acetyl CoA carboxylase (ACC) (Thermo, MA5-15025). Following overnight incubation with the primary antibody, blots were washed 3X with TBST and incubated with the secondary antibody, AlexaFluor 568 (1:2000, Donkey, anti-rabbit, Life Technologies, Eugene, OR) for 1–2 hours at room temperature with rocking. Western blot color images were inverted to show bands in black and background in white for easier visualization.

### Malonyl CoA ELISA

The provided manufacturer’s protocol was followed (Human Malonyl Coenzyme A ELISA kit, MyBioSource, San Diego, CA). Briefly, constructs were isolated as previously described, washed 3X with PBS, and lysed using RIPA buffer containing protease inhibitors. Lysates were isolated and 100μL was immediately added to the ELISA microplate and incubated for 2 hours at 37°C with rocking. Samples and standards were aspirated, and 100μL of Biotin-antibody was incubated in wells for 1 hour at 37°C. Wells were then washed 3X with provided wash buffer and then incubated with 100μL HRP-avidin for 1 hour at 37°C. Wash cycle was repeated and then TMB substrate (90μL) was added and incubated for 20 minutes in the dark. Stop solution (50μL) was added and plate was read immediately at 450nm with blank correction (BMG Labtech, Ortenberg, Germany). A 4-parameter curve and linear regression was used to calculate the concentrations of samples based on the standard curve.

### Matrix thickness measurements

3D constructs were isolated, washed 3X with PBS, and then fixed in 3% glutaraldehyde for 20 minutes. Constructs were blocked with 2% bovine serum albumin for 30 minutes and then stained with Alexa Fluor 546 phalloidin (Life Technologies) and DAPI for 1hr and 10 minutes, respectively. Stain was aspirated followed by washing 2X with PBS. Fixed constructs were added to microscope slides with 1 drop of glycerol added followed by the coverslip. Imaging was taken on the FV500 confocal microscope and z-stack was performed. Thickness of the matrix was determined by measuring the distance from the lowest cell layer to the top cell layer.

### Statistical analysis

GraphPad Prism 7.02 was used to determine statistical significance using a one-way or two-way ANOVA, where appropriate. A p≤0.05 was considered statistically significant. The n number for each experiment is listed in the appropriate figure legend. All graphs show mean ± standard error of the mean.

## Results and discussion

### Expression of hypoxia-associated proteins in HCFs and HKCs

**Fig 1:**
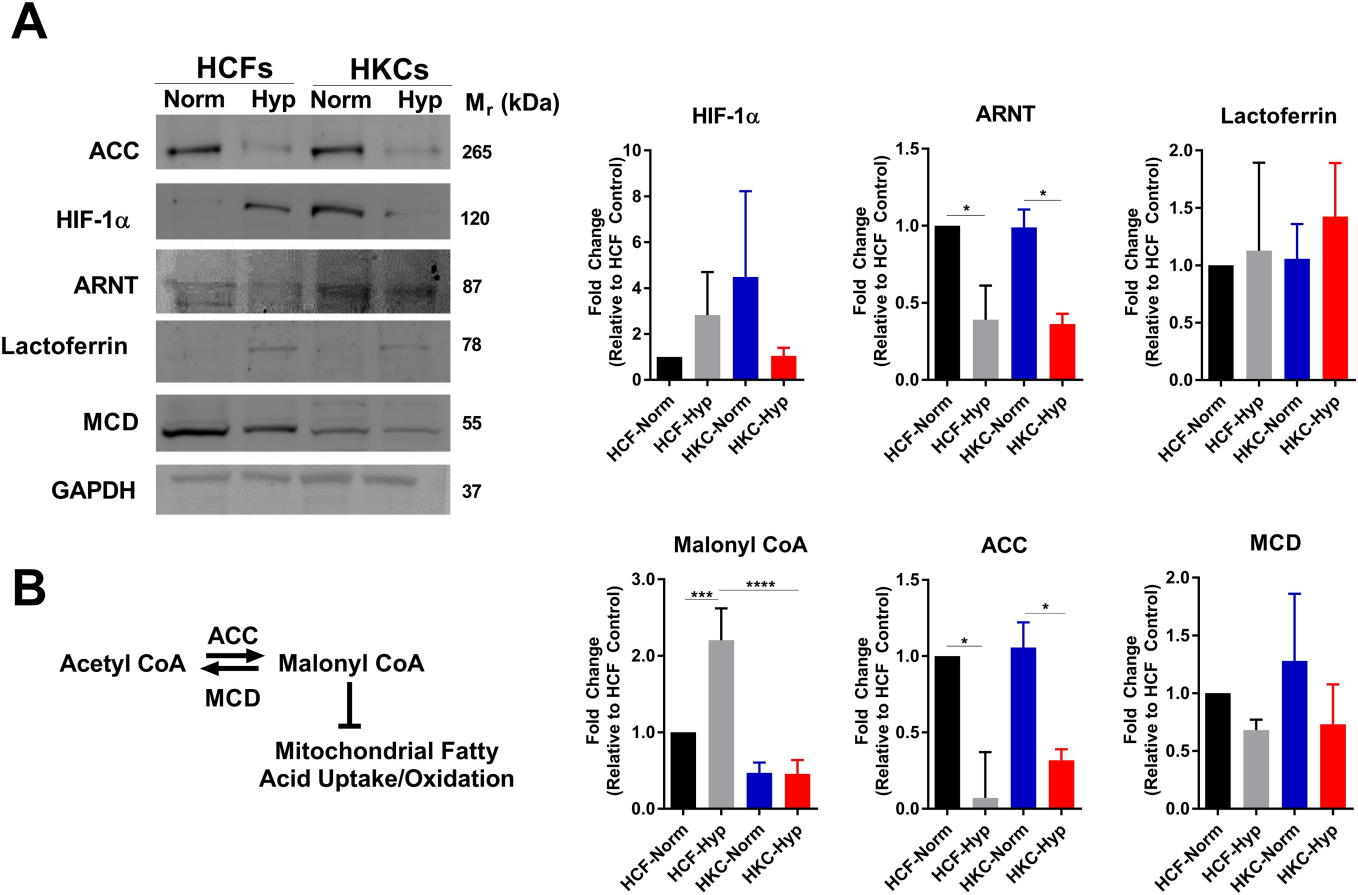
Regulation of hypoxia-inducible proteins and malonyl CoA levels by hypoxia. (A) Protein expression of hypoxia-inducible proteins, including HIF-1α, aryl hydrocarbon receptor nuclear transport (ARNT), and lactoferrin. (B) Schematic depicting regulation of malonyl CoA levels by acetyl CoA carboxylase (ACC) and malonyl CoA decarboxylase (MCD). Protein expression of ACC and MCD measured by Western blot with quantification determined using densitometry with background subtraction. The contrast and brightness were altered to 46% and -20%, respectively, uniformly throughout the ARNT blot in order to enable increased distinction of the band from the background. The unmodified, uncropped western blots are provided in the supplemental material. Malonyl CoA levels in HCFs and HKCs were measured by ELISA. All data was normalized to HCF-Normoxic control. n≥3, error bars represent SEM. An ANOVA was used to determine statistical significance with *p≤0.05, **p≤0.01, ***p≤0.001, ****p≤0.0001.

In order to determine endogenous levels and stability of hypoxia-associated proteins in HCFs and HKCs, we investigated HIF-1 protein expression in our 3D constructs following incubation in normoxic or hypoxic environments. Hypoxia led to a slight increase, though not statistically significant, in HIF-1α stabilization in HCFs with no further increase in HKCs (Fig 1A). Aryl hydrocarbon nuclear translocator (ARNT), is a binding partner to HIF-1α and was found to be reduced 2-fold (p≤0.05) following hypoxia induction suggesting prolonged oxygen deficiency may affect stability of cytosolic ARNT (Fig 1A). The iron-binding protein, lactoferrin, has been shown to be protective against oxidative stress following hypoxia in the corneal epithelium *in vitro* [26]. We found no change in lactoferrin expression in HCFs and HKCs upon hypoxia induction suggesting its expression may be differentially regulated in fibroblasts compared to epithelial cells (Fig 1A).

**Fig 2:**
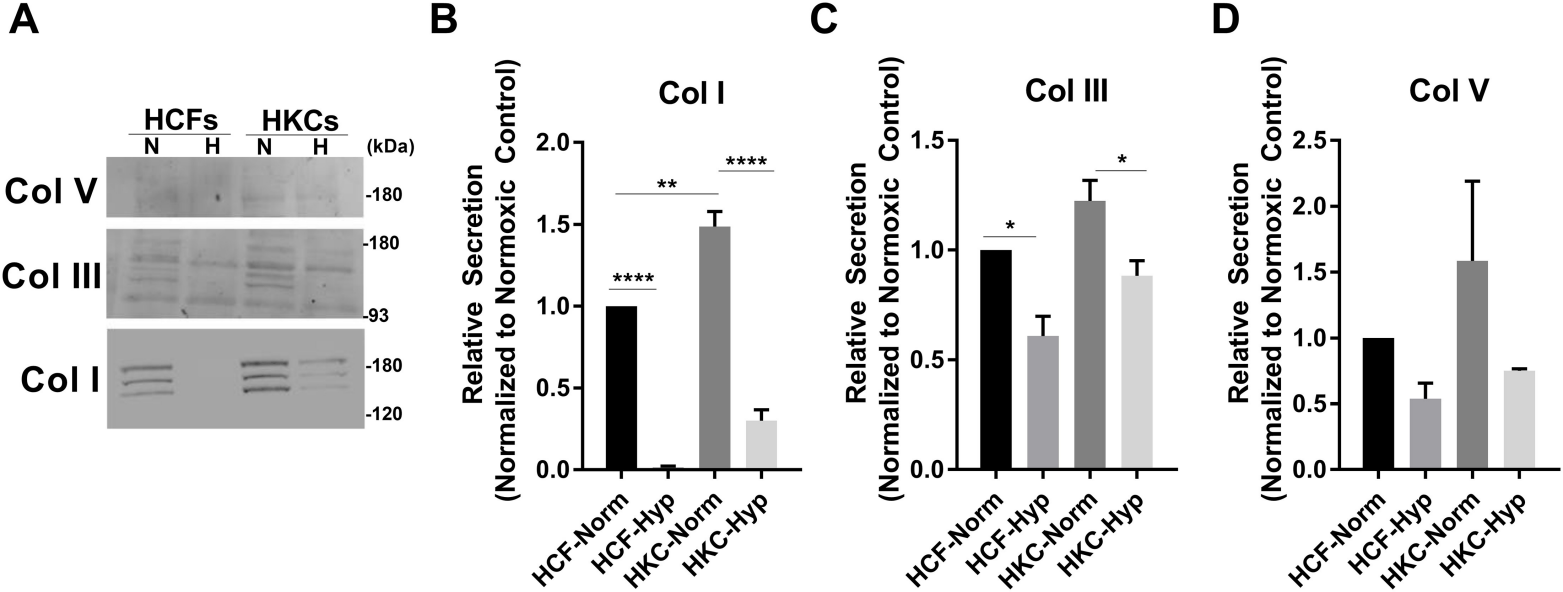
Secretion of the major collagens, Col I, III, and V, detected in the media following exposure to normoxic or hypoxic (2% O_2_) conditions. (A) Representative western blots and (B-D) quantification by densitometry showing a reduction in Col I in both HCFs and HKCs following hypoxia exposure. Error bars represent standard error of the mean. n = 3 for Col I and III, n = 2 for Col V. A two-way ANOVA was used to determine statistical significance with *p≤0.05, **p≤0.01, ***p≤0.001, and ****p≤0.0001.

Malonyl CoA is a factor responsible for inhibiting mitochondrial lipid oxidation and is associated with reducing oxidative stress [27–29]. In order to determine the effects of hypoxia on malonyl CoA, we measured levels by ELISA in HCFs and HKCs at normoxic and hypoxic conditions (Fig 1B). We found a reduction in HKCs at normoxia (2.1-fold) compared to HCFs with hypoxia increasing malonyl CoA levels (2.2-fold, p≤0.001) in HCFs alone. Lower malonyl CoA levels in HKCs in normoxic conditions may correlate with the increased oxidative stress associated with KC in numerous studies [19, 30–32] (Fig 1B). Regulation of malonyl CoA flux is determined by the expression and activity of the enzymes ACC and MCD, which coordinate the reversible reaction of acetyl CoA to malonyl CoA. ACC expression was reduced (3-fold, p≤0.05) in HCFs and HKCs with hypoxia-induction with no significant changes in MCD levels (Fig 1B). Given that ACC expression was reduced with hypoxia, it is possible that a reduction in lipid biosynthesis also occurs with lower utilization of malonyl CoA for fatty acid biosynthesis resulting in the observed increase in malonyl CoA. While hypoxia led to an increase in malonyl CoA levels in HCFs, we saw no such increase in HKCs which may be a result of reduced ACC levels during hypoxia (Fig 2B), as well as changes in lipid metabolism or defects in recognition of hypoxia-induced oxidative stress.

### Effects of hypoxia on matrix secretion

Collagen I, III, and V are the primary isoforms secreted by corneal stromal fibroblasts, which mediate assembly of the surrounding ECM. In order to determine the effects of hypoxia on ECM secretion, we measured Collagen I, III, and V levels in the media in normoxic and hypoxic conditions by Western blot (Fig 2A). We found significant reduction in Collagen I secretion in both HCFs and HKCs (p≤0.0001) following induction of hypoxia (Fig 2B). HKCs showed a significant decrease in Collagen III while Collagen V secretion was not affected by hypoxia in either cell type (Fig 2C and 2D). We did detect three bands present in the Collagen I and III western blots from the media compared to the expected two for Collagen I representing the two predominant splice variants. It is unclear the source of the additional bands but may be associated with partially crosslinked forms or cross-reactivity of the antibody. Our results showed sensitivity to hypoxia by HCFs and HKCs with a profound reduction in Collagen I secretion following hypoxia exposure, which is the major structural component within the corneal stroma.

### MMPs and hypoxia

**Fig 3:**
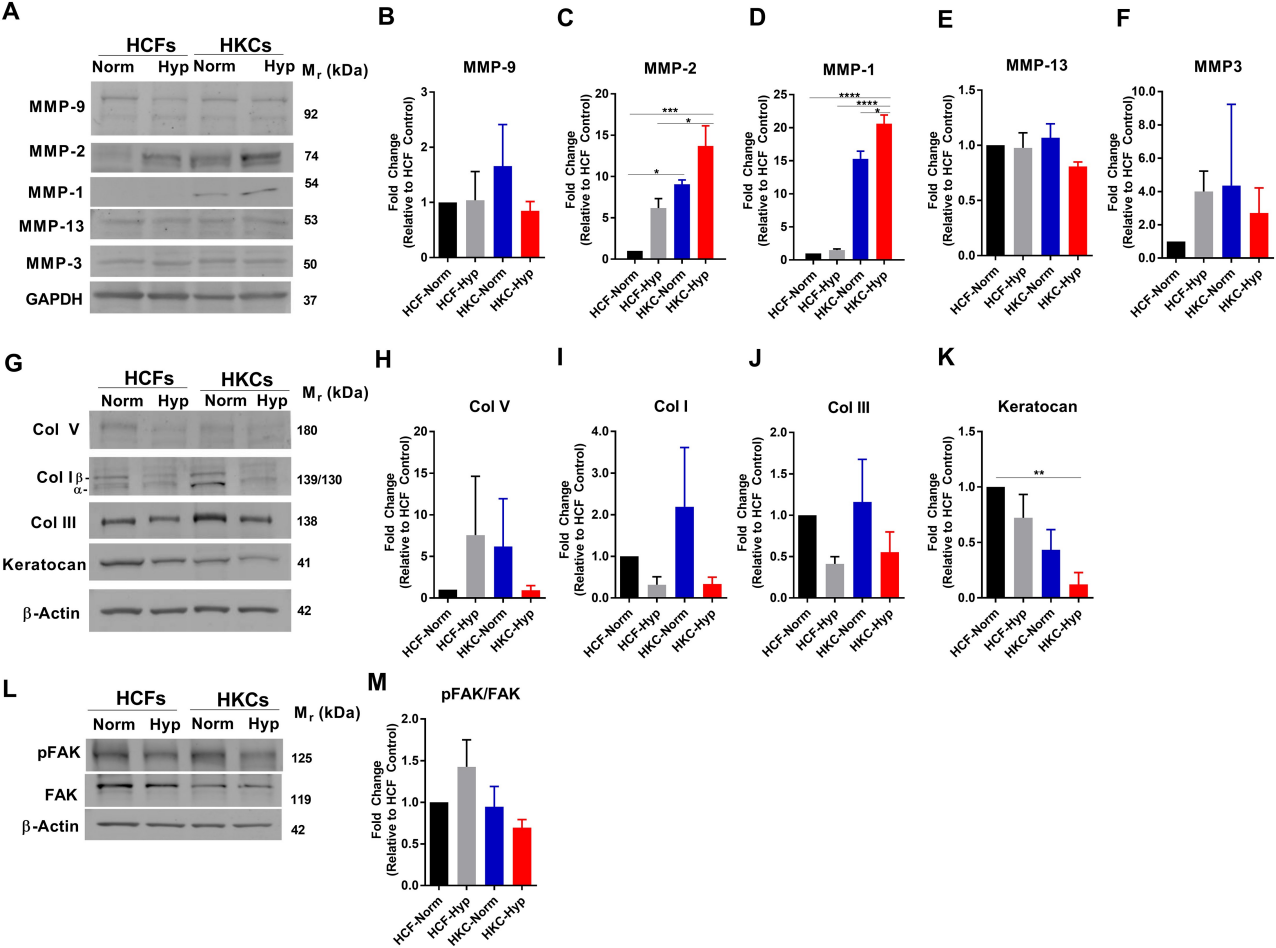
Effects of hypoxia on MMP, collagen expression, and FAK signaling in HCFs and HKCs under normoxic or hypoxic (2% O_2_) conditions. (A) Representative western blots and (B-F) quantification for MMP-9, MMP-2, MMP-1, MMP-13, and MMP-3 expression. (G) Representative western blots of cytosolic collagen and keratocan expression and quantification of (H-K) Collagens I, III, V, and keratocan expression. (L) Representative western blot of pFAK and FAK expression and (M) quantification of the ratio of pFAK/FAK. Quantification determined using densitometry. n = 3, statistical significance determined by an ANOVA with * = p≤0.05, ** = p≤0.01, *** = p≤0.001, and **** = p≤0.0001.

MMPs have been shown to play integral parts in remodeling of the ECM following injury by promoting degradation and removal of excess collagen in order to reform the native matrix structure [33, 34]. This remodeling process is essential for maintaining a transparent central cornea, which is required for proper vision. In our study, we measured increased MMP-1 and -2 expression (15-fold and 10-fold, p≤0.05, respectively) by HKCs at normoxic conditions compared to HCFs, which was further increased upon hypoxia induction (Fig 3A–3C). Hypoxia significantly upregulated MMP-2 expression by 6-fold in HCFs suggesting a direct link to activation of ECM remodeling and stabilization of HIF-1α (p≤0.05, Fig 3C). This increase in MMP expression in HKCs correlates with previous studies reporting elevated MMP activity in KC corneal buttons [35, 36]. Though the role of MMP activity in KC progression is still unclear, our study suggests that hypoxia regulates expression of MMP-1 and MMP-2 in HKCs suggesting that HIF-1 may play a direct role in regulating genes associated with ECM remodeling.

Since we measured reduced Collagen I secretion by HCFs and HKCs following hypoxia-induction (Fig 2), we sought to verify that reduced collagen secretion correlated with lower cytosolic expression. Both HCFs and HKCs showed slight reductions in cytosolic Collagen I levels following 1 week in hypoxia with a 2-fold reduction in HKCs (Fig 3G and 3H). Collagen III expression was also affected by hypoxia with decreases in both HCFs and HKCs (Fig 3I) in agreement with slight reductions secreted into the media (Fig 2). In agreement with reduced Collagen I expression and elevated MMP-1 and -2 expression by HCFs and HKCs, we measured reduced ECM thickness based on the z-stack profile collected by confocal microscopy in both HCFs and HKCs (Table 1). Interestingly, both HCFs and HKCs assembled a thinner ECM following 1 week in hypoxia suggesting a role for increased MMP activity in addition to altered ECM secretion.

**Table 1:** ECM thickness after 4 weeks at normoxic conditions or maintained for 3 weeks at normoxia and then transferred to a hypoxia environment (2% O_2_) for 1 week. The distance from top to bottom cell layer was measured by confocal microscopy. n = 3, mean±S.E.M. Statistical significance determined by ANOVA, comparing all values to HCF-Normoxic controls.

Cell Type	ECM Thickness (microns)	Significance
HCF-Normoxic	79±1.0	-
HCF-Hypoxic	59.17±3.82	**p≤0.01
HKC-Normoxic	66.73±2.83	n.s.
HKC-Hypoxic	57.33±2.52	**p≤0.01

We have previously reported that HKCs show elevated α-SMA and Collagen III expression compared to HCFs correlating with myofibroblast differentiation in the KC cornea [24]. Studies in corneal wound healing have shown a downregulation in the small leucine-rich proteoglycan, keratocan, upon myofibroblast differentiation from keratocytes [37, 38]. We sought to determine if hypoxia alone influenced keratocan expression in HCFs and HKCs. The expression of keratocan was lower in HKCs at both normoxic and hypoxic conditions (p≤0.001) compared to HCFs suggesting that HKCs have altered proteoglycan expression that may affect matrix structure (Fig 3D and 3F). Furthermore, we did not measure any significant changes in keratocan expression with hypoxia-induction.

Focal adhesion kinase (FAK) is an important mediator of matrix-cell signaling with the activation of downstream elements that regulate fundamental cell processes required for survival, proliferation, and migration [39, 40]. Since hypoxia reduced ECM secretion and expression, we sought to determine if FAK signaling was affected which might lead to downstream effects on cell signaling. Interestingly, we found no significant effect on the ratio of activated phosphorylated FAK to FAK levels suggesting that this pathway may be exempt from the immediate effects of hypoxia (Fig 3L and 3M).

## Discussion

The avascular nature of the cornea requires oxygen supply from the external surface via the tear film or from the posterior aqueous humor. Contact lenses are commonly prescribed to KC patients to correct for the astigmatism induced by the irregular-shaped corneal surface [41, 42]. Studies have shown that contact lenses, specifically scleral lenses, reduce oxygen levels that reach the corneal surface thereby contributing to localized hypoxia [12, 43]. Acute effects of hypoxia on the corneal surface are associated with corneal edema [44, 45] and changes in osmolarity [46]. More chronic, sustained effects of hypoxia on corneal thickness, independent of edema, are relatively unknown. Given that the cornea also plays a fundamental role in providing for the majority of the refractive power of the eye, changes in ECM structure within the stroma can lead to substantial effects on visual acuity. In the context of KC, thinning of the corneal stroma is a key clinical feature of the disease that leads to the bulging phenomena of the central apex and development of an irregular astigmatism. KC has been associated with increased Collagen III and fibronectin deposition within the corneal stroma correlating with scar development [47]. Studies have also reported alterations present in KC corneal buttons in the epithelial-stromal interface separated by the basement membrane [48, 49]. To-date, the cause of central corneal thinning remains elusive. Early studies of KC identified elevated MMP expression both *in vitro* and *ex vivo* suggesting that ECM degradation, rather than solely reduced ECM assembly, may promote KC progression [35, 50–53]. However, the direct relationship between upregulated MMP expression and stromal thinning in the context of KC has not been established. A number of studies have identified that HKCs are more susceptible to oxidative stress which may suggest that reduced ECM expression or keratocyte apoptosis are the primary drivers of stromal thinning [30, 54, 55]. We have previously shown that HKCs exhibit increased oxidative stress and favor aerobic glycolysis and lactate production with reduced citric acid cycle intermediates compared to HCFs even at sufficient oxygen levels [19]. These data suggest that HKCs are less reliant on oxidative phosphorylation for energy production. Hypoxia results in reduction of the terminal electron acceptor (O_2_) and therefore may increase the oxidative stress within a cell with the lack of a sufficient terminal electron acceptor required during oxidative phosphorylation. A cell’s counter-mechanism to enable survival during hypoxia is to stabilize HIF-1α, which then upregulates aerobic glycolysis to generate ATP for the continual energy demand [16, 17, 20].

Our current study investigated whether HKCs are inherently more susceptible to hypoxia-induced oxidative stress. Two possibilities regarding the response to hypoxia in KC seem plausible: 1) Since HKCs have inherent oxidative stress even at normoxic conditions, they are more susceptible to hypoxia-induced cell stress that may affect ECM secretion, expression, and deposition, or 2) Given that HKCs show increased lactate levels even at normoxic conditions, they are less dependent on oxidative phosphorylation for ATP production and therefore will be unaffected with a reduction in oxygen levels. Our results suggest that the first scenario is most likely, given that an immediate reduction in Collagen I secretion and increased MMP expression was found post-induction of hypoxia. In addition to increased MMP-1 and -2 expression, we identified lower keratocan expression in HKCs both at normoxia and hypoxia. Keratocan knockout in mice has been correlated with thinner corneal structure and disorganized collagen fibril deposition [56] suggesting that the altered keratocan expression detected in HKCs may contribute to inherent defects in ECM assembly that promote corneal structural defects. Indeed, we identified that hypoxia induction resulted in assembly of a thinner ECM in both HCFs and HKCs. It is possible that MMP upregulation following induction of hypoxia may contribute to enzymatic degradation of the stroma in KC. Limitations of our study did not evaluate activity of detected MMPs in order to determine if MMP-1 or -2 may contribute to increased ECM degradation following hypoxia. Furthermore, additional studies testing the effects of chronic hypoxia at less severe oxygen levels (10–15%) may be required in order to associate contact lens use with changes in ECM deposition in the context of KC. Collectively, our study revealed that acute hypoxia influences ECM structure in corneal fibroblasts with modulation of MMP expression and collagen secretion and identified a differential response in HKCs that may be related to differences in basal metabolic rates reported in previous studies [19, 32]. Further studies are warranted to determine if treatment modalities that lead to reduced oxygen levels within the corneal stroma affect ECM deposition and degradation in KC *in vivo*.

## Supporting information

S1 Fig Schematic of experimental design for hypoxia induction. 3D cultures were maintained at normoxic conditions for 3 weeks and then transferred to a hypoxic environment (2% O_2_) or maintained at normoxia for 1 week for treatment group or control, respectively.
(TIF)

S2 Fig Effect of exogenous oxygen levels on pH of media in 3D cultures of HCFs in normoxic and hypoxic conditions (2% O2). A significant reduction in pH of the media occurs at 48 hours post-hypoxia induction. Maximum, minimum, and the interquartile range are shown. n = 3, significance determined by ANOVA.
(TIF)

S3 Fig Compiled western blot data showing uncropped, unmodified images. (PDF)
