# Supplementary material for: Acute hypoxia influences collagen and matrix metalloproteinase expression by human keratoconus cells in vitro
Source: PLoS One. 2017 Apr 20;12(4):e0176017. doi: 10.1371/journal.pone.0176017 (PMC5398580; doi:10.1371/journal.pone.0176017)
Supplement: S3 Fig — (PDF) [file pone.0176017.s003.pdf]

ACC

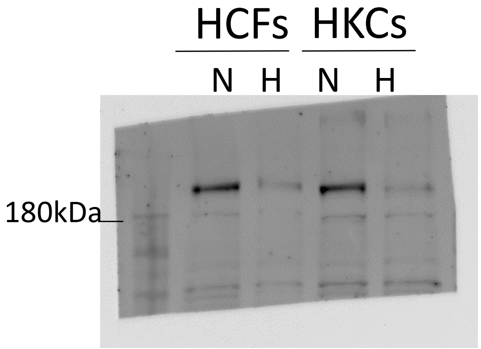

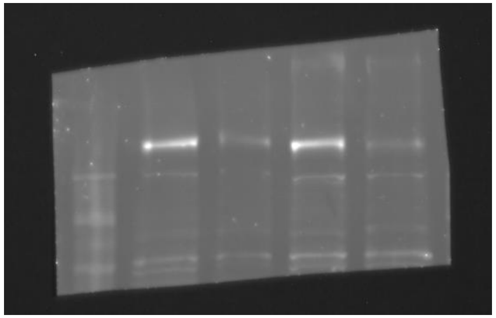

Uncropped,
non-inverted
blot

Uncropped western blot of protein isolated from HCFs and HKCs following hypoxia exposure. Blot was cut following blocking and prior to incubation with primary antibody.

HIF-1α

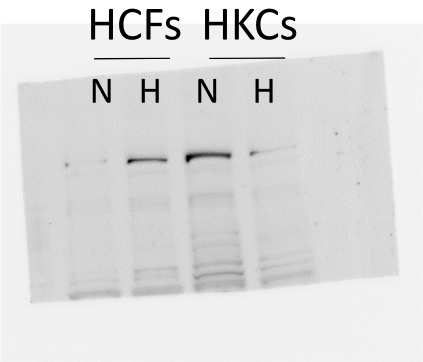

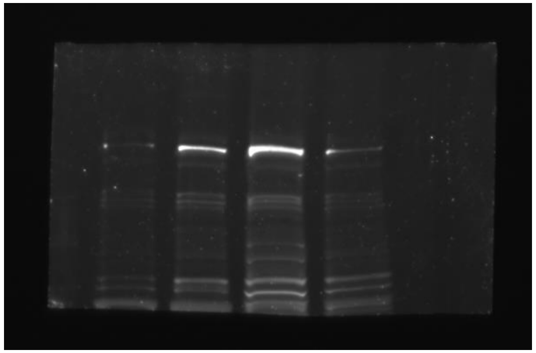

Uncropped,
non-inverted
blot

Uncropped western blot of protein isolated from HCFs and HKCs following hypoxia exposure. Blot was cut following blocking and prior to incubation with primary antibody.

ARNT

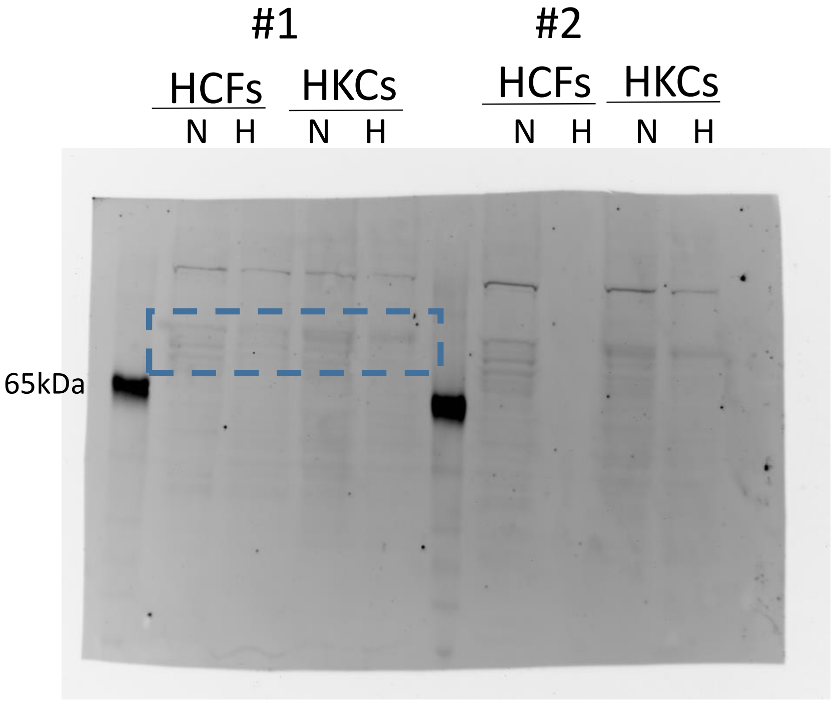

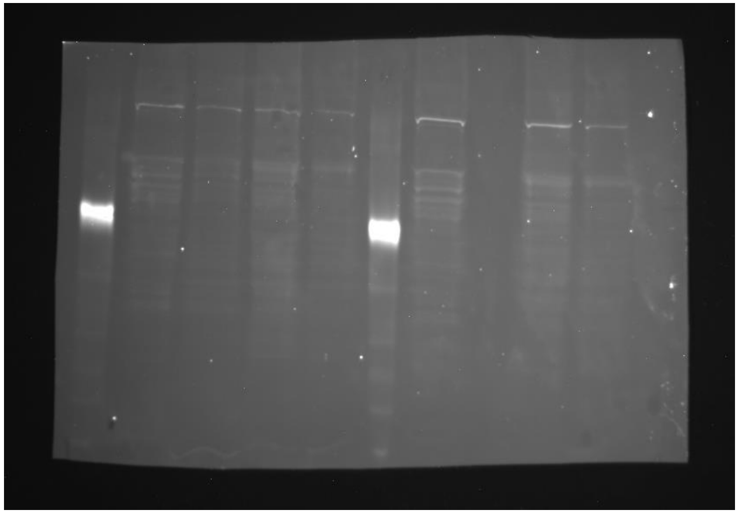

Uncropped,
non-inverted
blot

Uncropped blots of protein isolated from HCFs and HKCs following hypoxia exposure. Dashed blue box denotes cropped area shown in Fig 1A. Blot was cut following blocking and prior to incubation with primary antibody.

MCD

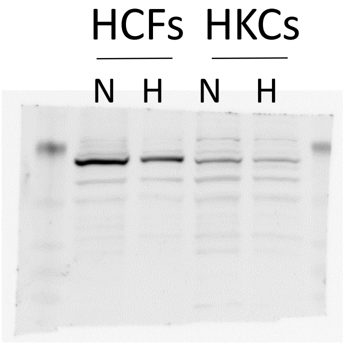

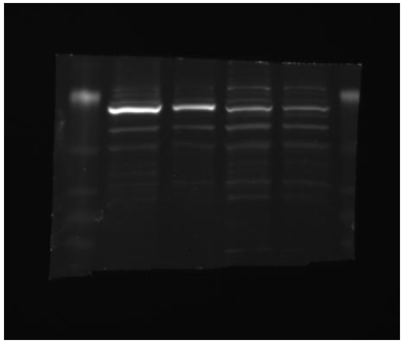

Uncropped, non-inverted blot

Uncropped western blot of protein isolated from HCFs and HKCs following hypoxia exposure. Blot was cut following blocking and prior to incubation with primary antibody.

Lactoferrin

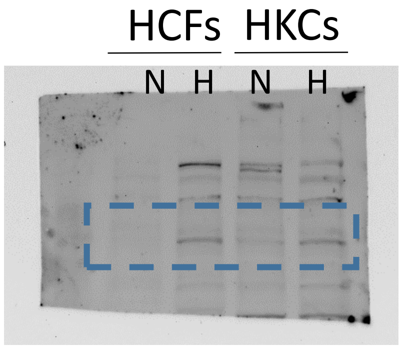

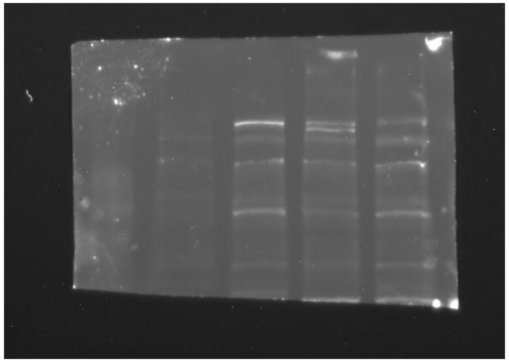

Uncropped, non-inverted blot

Uncropped blots of protein isolated from HCFs and HKCs following hypoxia exposure. Dashed blue box denotes cropped area shown in Fig 1A. Blot was cut following blocking and prior to incubation with primary antibody.

GAPDH

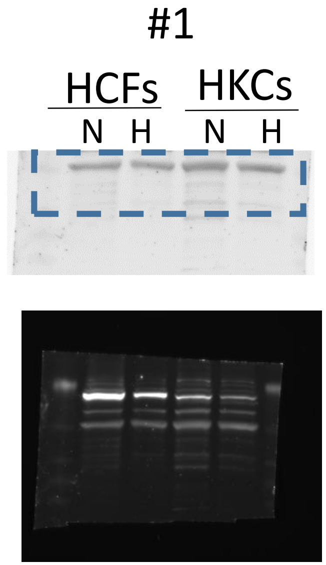

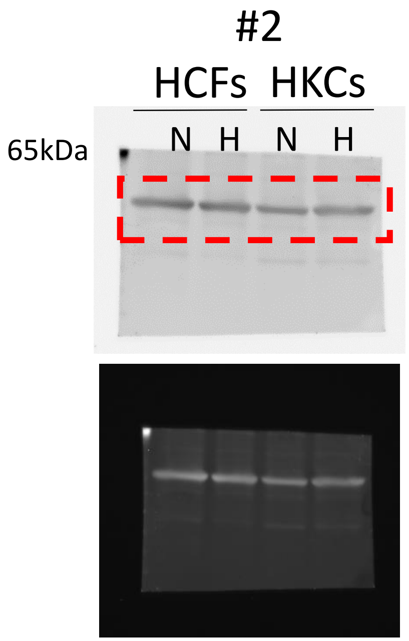

Uncropped blots of protein isolated from HCFs and HKCs following hypoxia exposure. Dashed blue box denotes cropped area shown in Fig 1A and dashed red box denotes cropped area shown in Fig 3A. Blot was cut following blocking and prior to incubation with primary antibody.

Collagen I

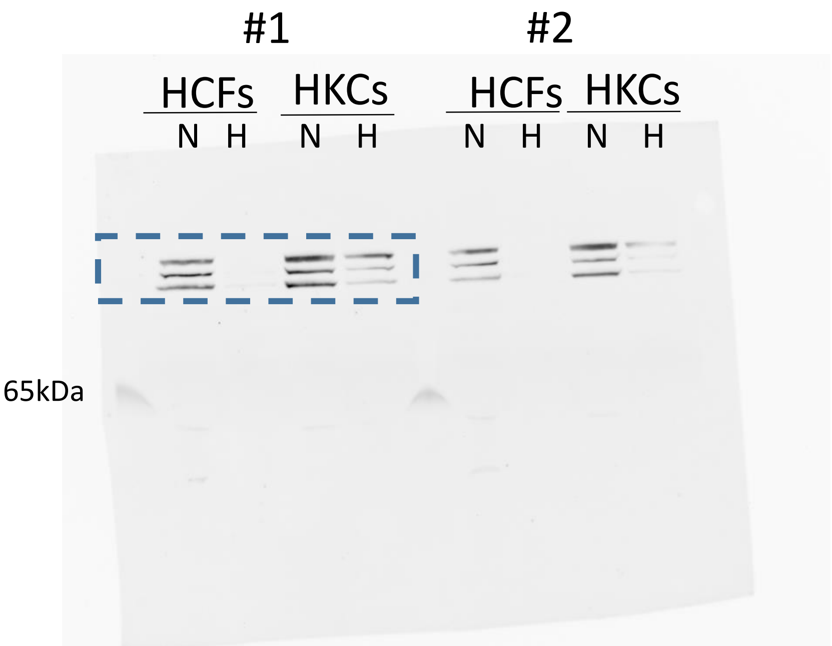

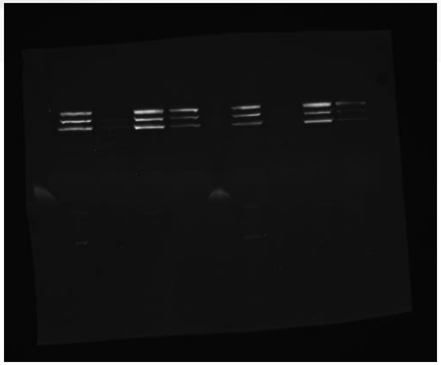

Uncropped,
non-inverted
blot

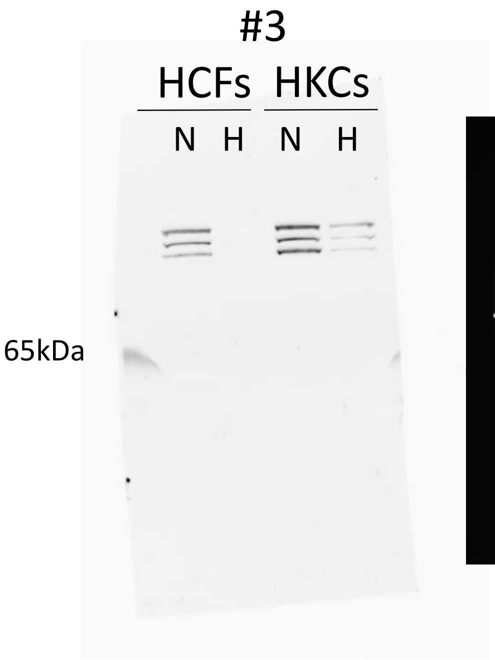

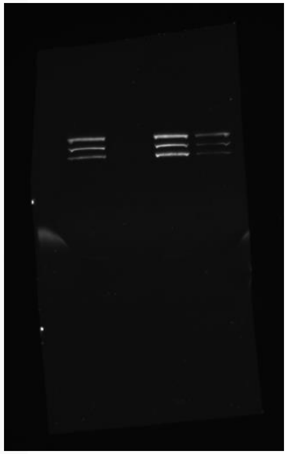

Uncropped,
non-inverted
blot

Uncropped blots of media isolated from HCFs and HKCs following hypoxia exposure for 3 replicates. Dashed blue box denotes cropped area shown in Fig 2.

Collagen III

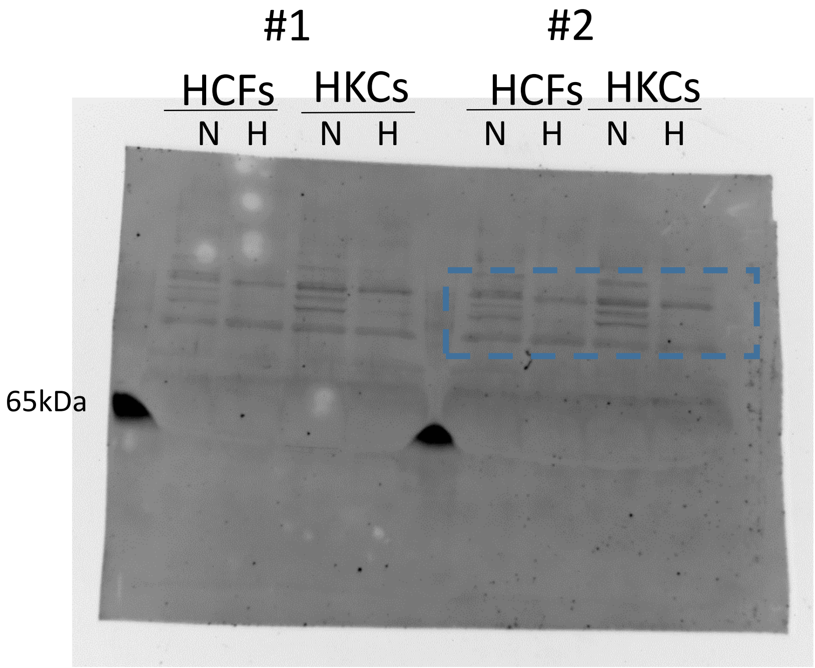

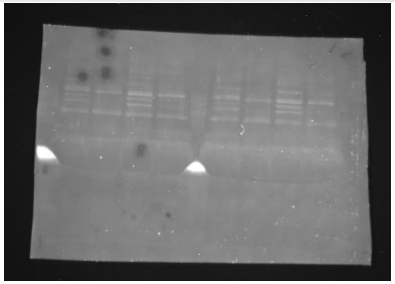

Uncropped, non-inverted blot

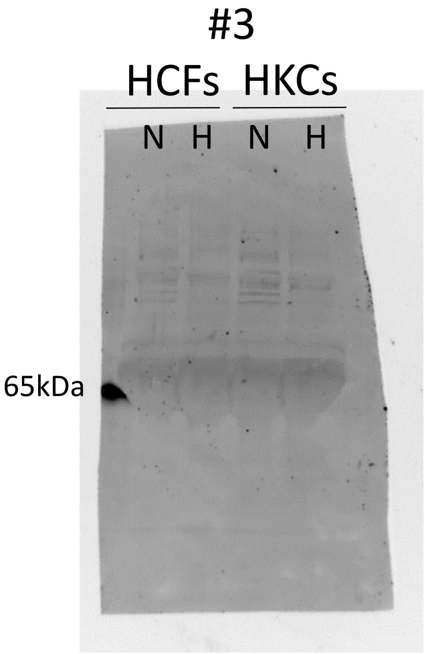

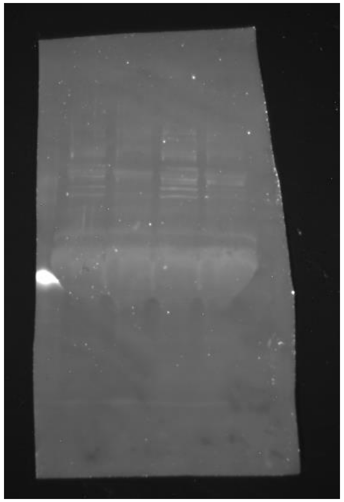

Uncropped, non-inverted blot

Uncropped blots of media isolated from HCFs and HKCs following hypoxia exposure for 3 replicates. Dashed blue box denotes cropped area shown in Fig 2. Large non-specific binding at 65kDa likely corresponds to albumin, a protein found in media containing FBS.

Collagen V

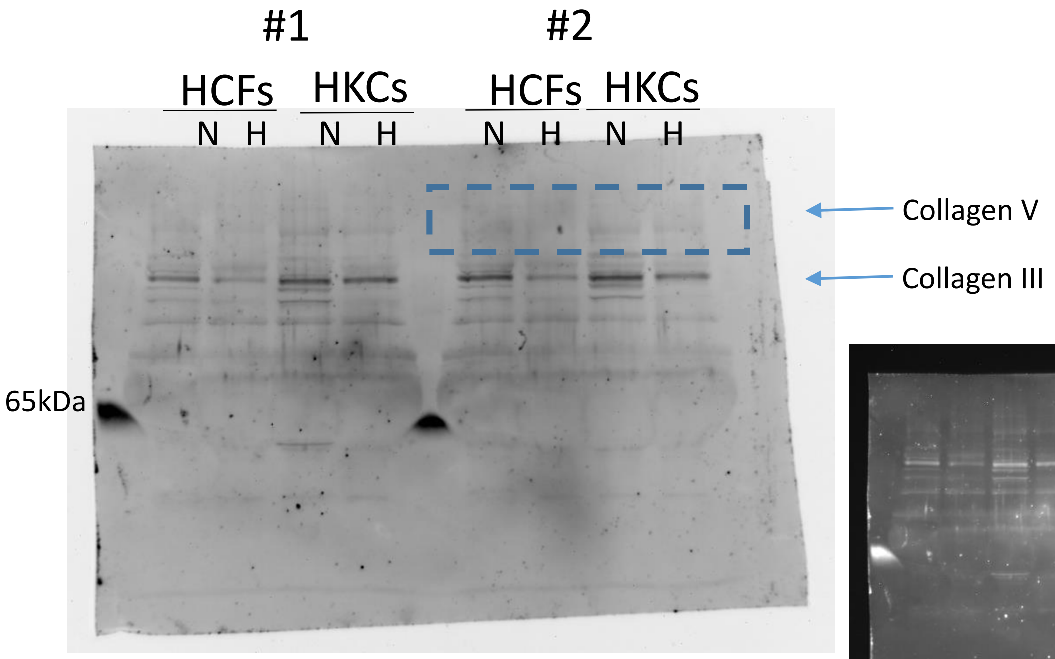

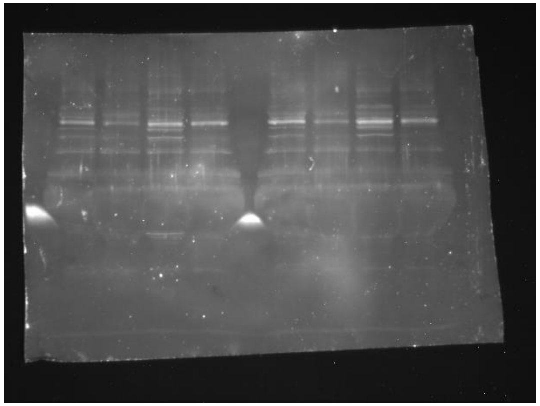

Uncropped, non-inverted blot

Uncropped blots of media isolated from HCFs and HKCs following hypoxia exposure for 2 replicates. Collagen III blot shown on previous page was re-probed with Collagen V antibody. Dashed blue box denotes cropped area shown in Fig 2.

MMP-9

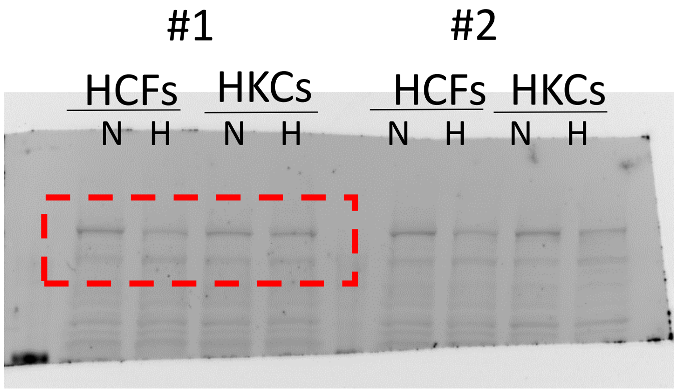

Uncropped, non-inverted blot

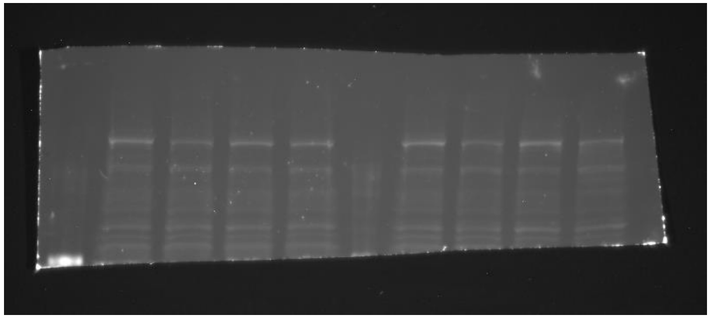

Uncropped blots of protein isolated from HCFs and HKCs following hypoxia exposure. Dashed red box denotes cropped area shown in Fig 3A.

MMP-2

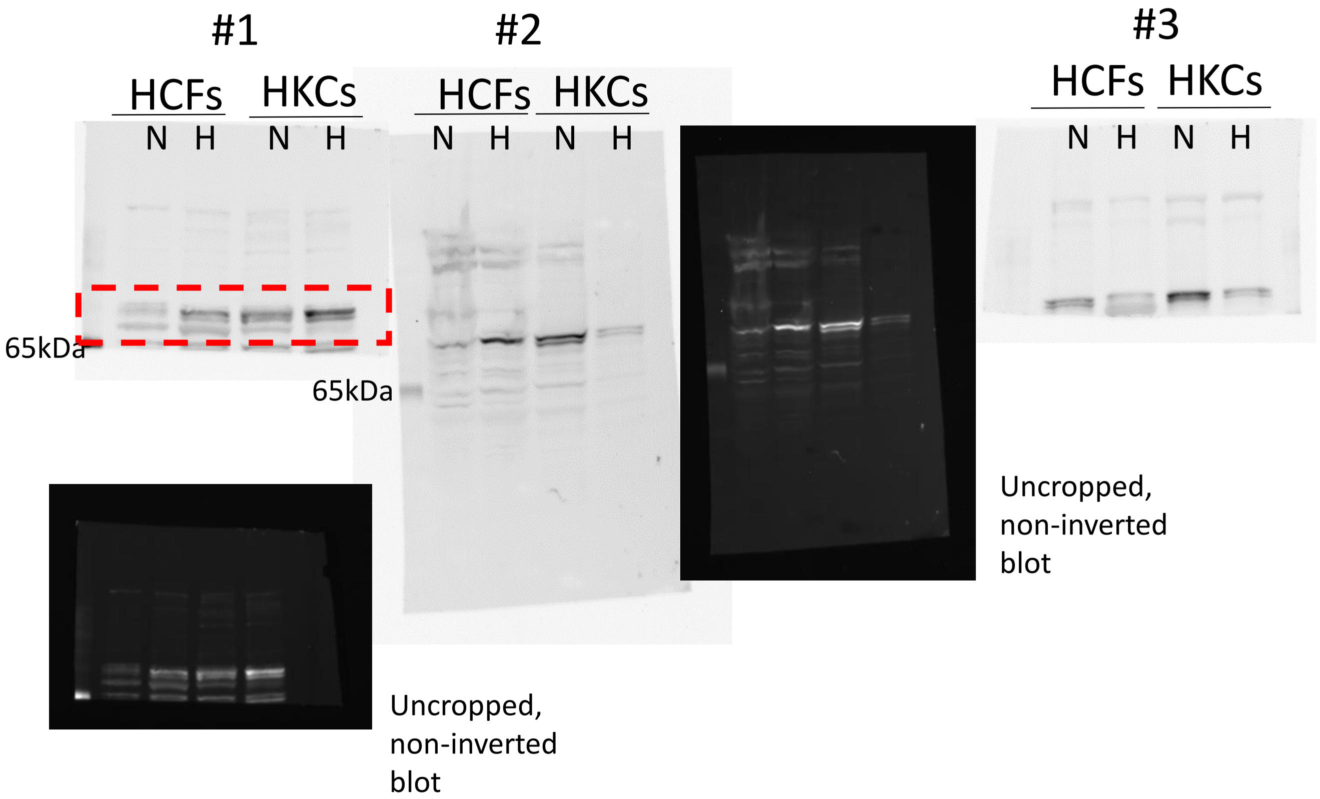

Uncropped blots of protein isolated from HCFs and HKCs following hypoxia exposure for 3 replicates. Dashed red box denotes cropped area shown in Fig 3A.

MMP-1

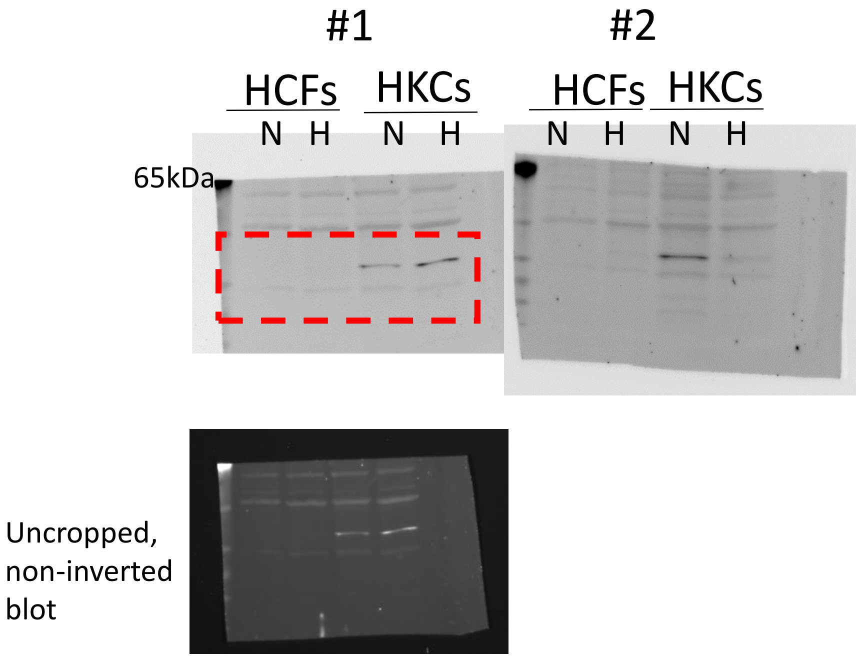

Uncropped blots of protein isolated from HCFs and HKCs following hypoxia exposure. Dashed red box denotes cropped area shown in Fig 3A.

MMP-13

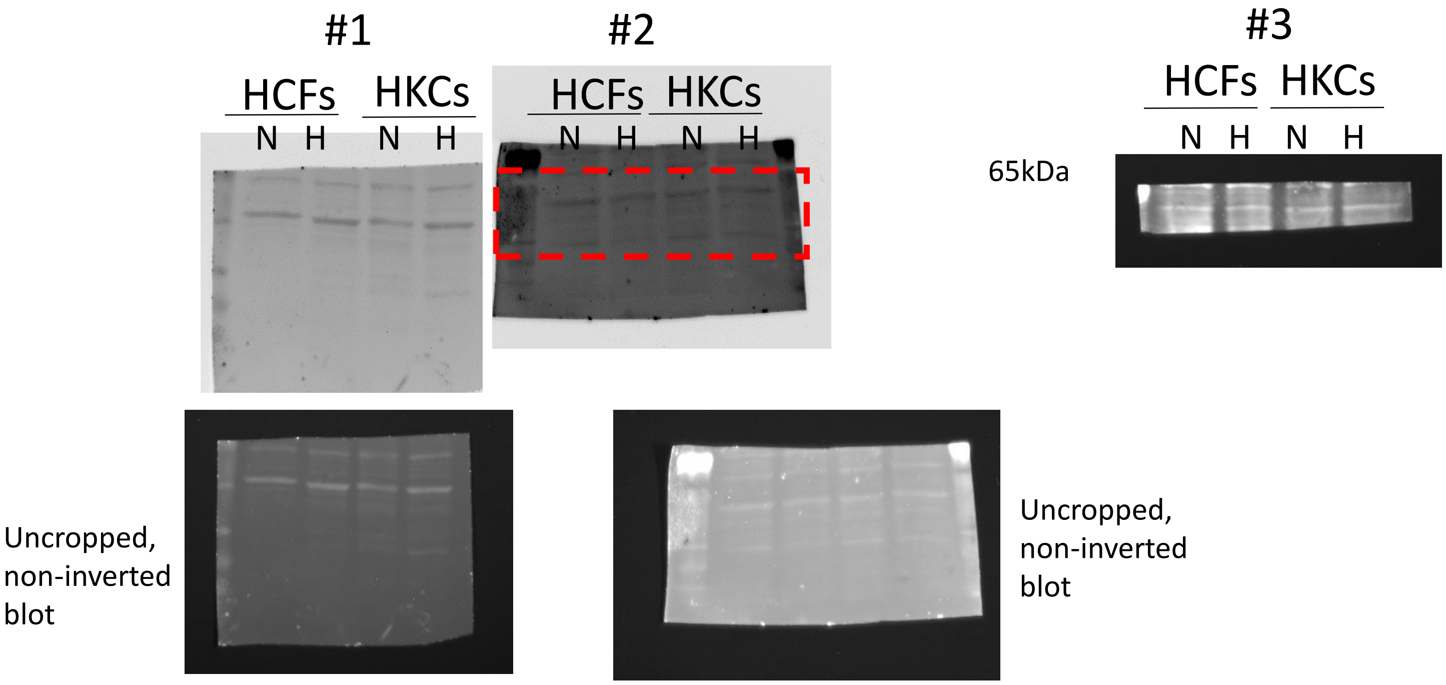

Uncropped blots of protein isolated from HCFs and HKCs following hypoxia exposure for 3 replicates. Dashed red box denotes cropped area shown in Fig 3A.

MMP-3

#1

HCFs HKCs

N H N H

65kDa

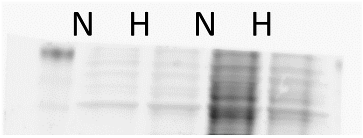

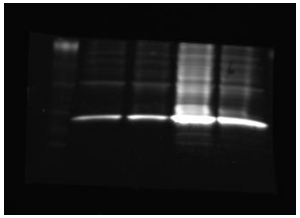

Uncropped,
non-inverted
blot

#2

HCFs HKCs

N H N H

65kDa

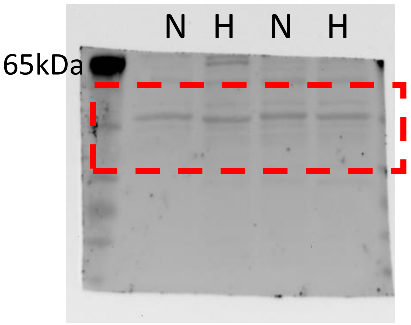

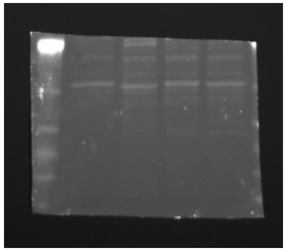

Uncropped,
non-inverted
blot

Uncropped blots of protein isolated from HCFs and HKCs following hypoxia exposure. Dashed red box denotes cropped area shown in Fig 3A.

Col V

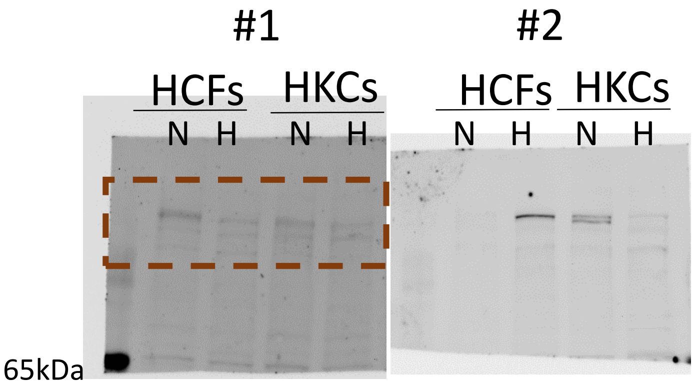

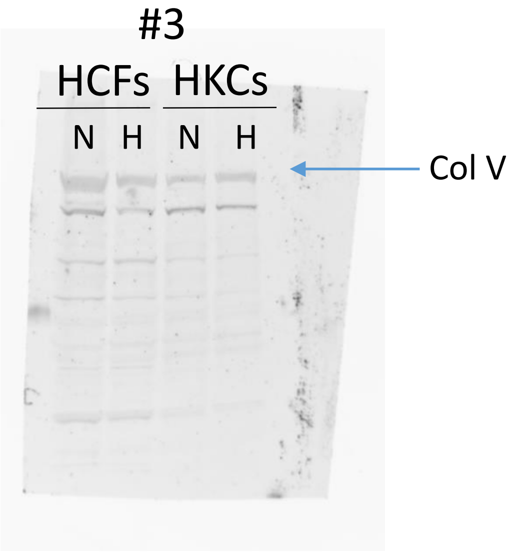

Uncropped, non-inverted blots

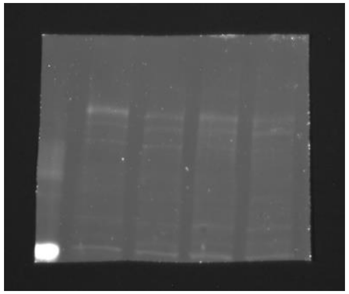

Uncropped blots of protein isolated from HCFs and HKCs following hypoxia exposure for 3 replicates. Dashed brown box denotes cropped area shown in Fig 3G.

Col I

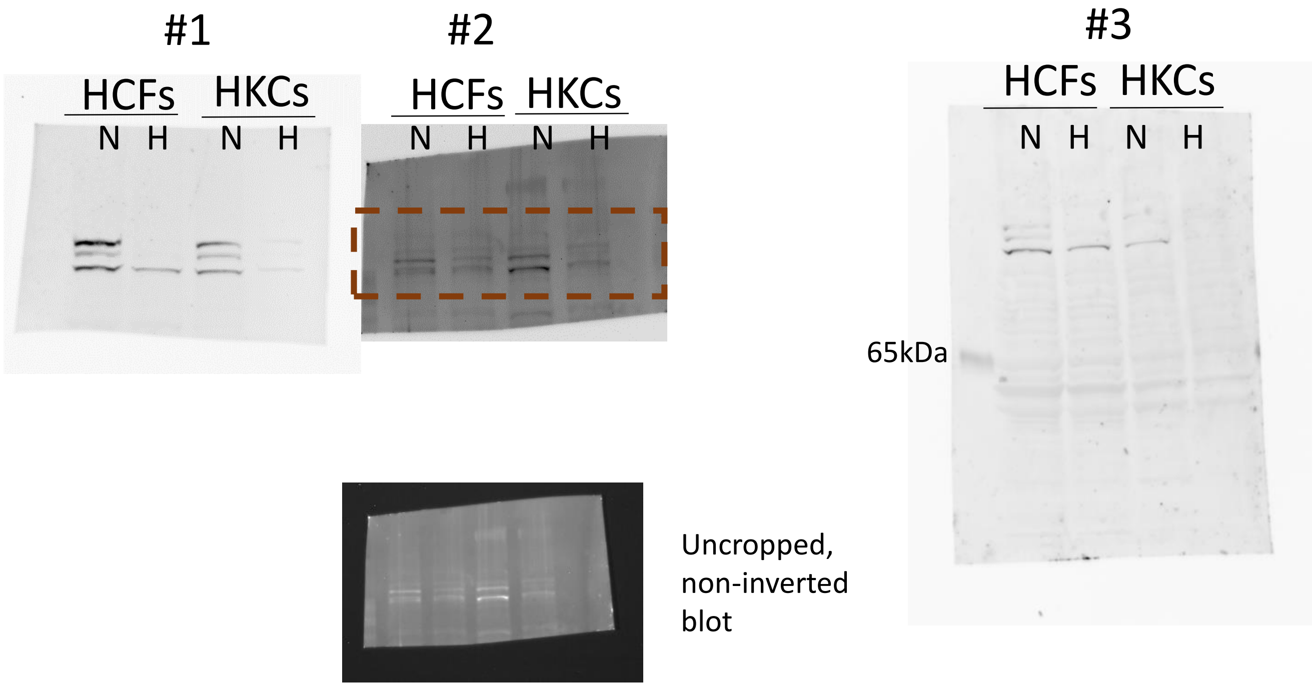

Uncropped blots of protein isolated from HCFs and HKCs following hypoxia exposure for 3 replicates. Dashed brown box denotes cropped area shown in Fig 3G.

Col III

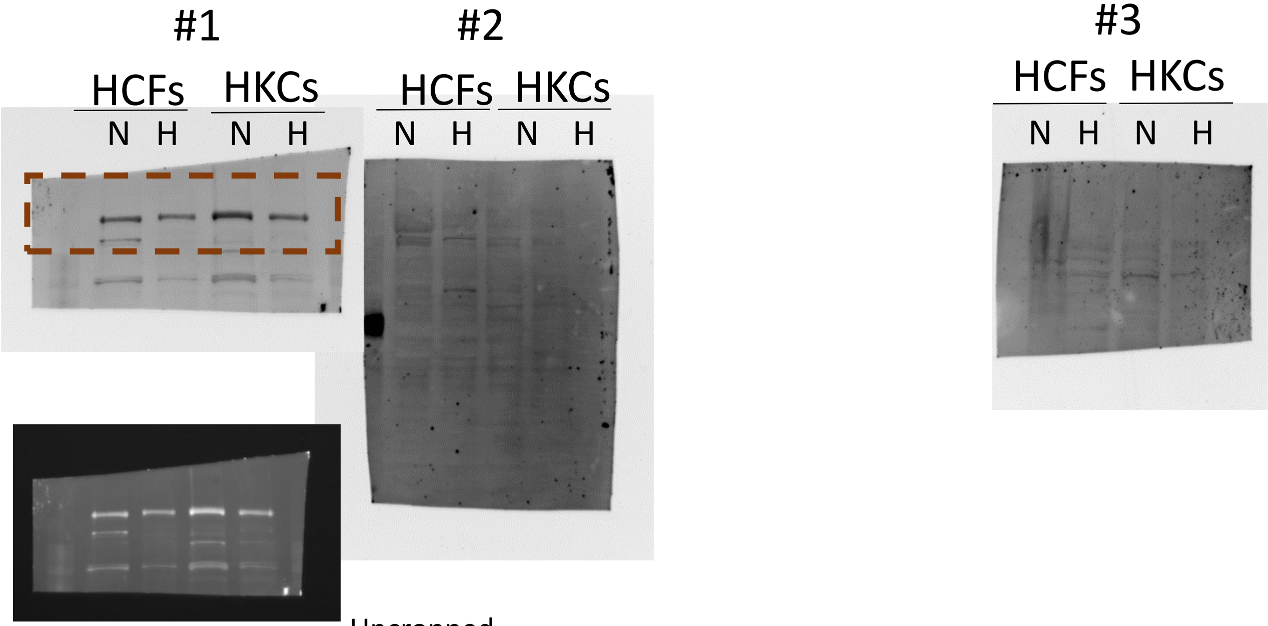

Uncropped,
non-inverted
blot

Uncropped blots of protein isolated from HCFs and HKCs following hypoxia exposure for 3 replicates. Dashed brown box denotes cropped area shown in Fig 3G.

Keratocan

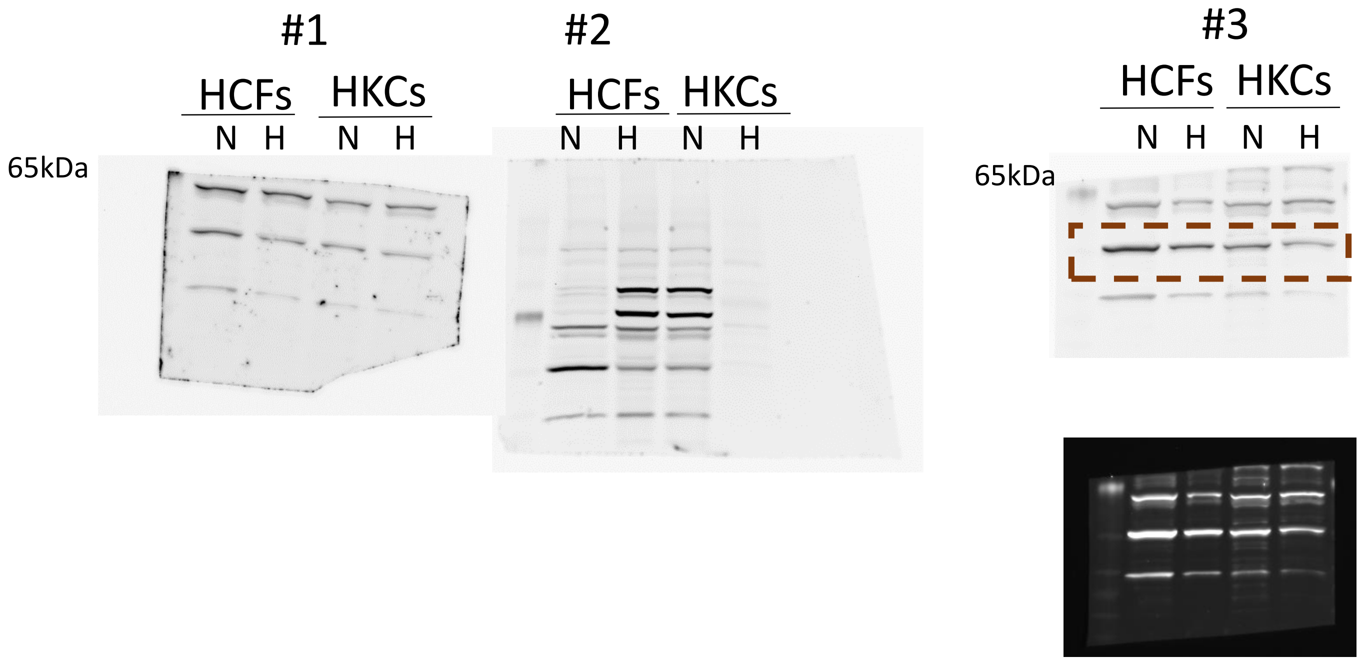

Uncropped, non-inverted blot

Uncropped blots of protein isolated from HCFs and HKCs following hypoxia exposure for 3 replicates. Dashed brown box denotes cropped area shown in Fig 3G.

β-Actin

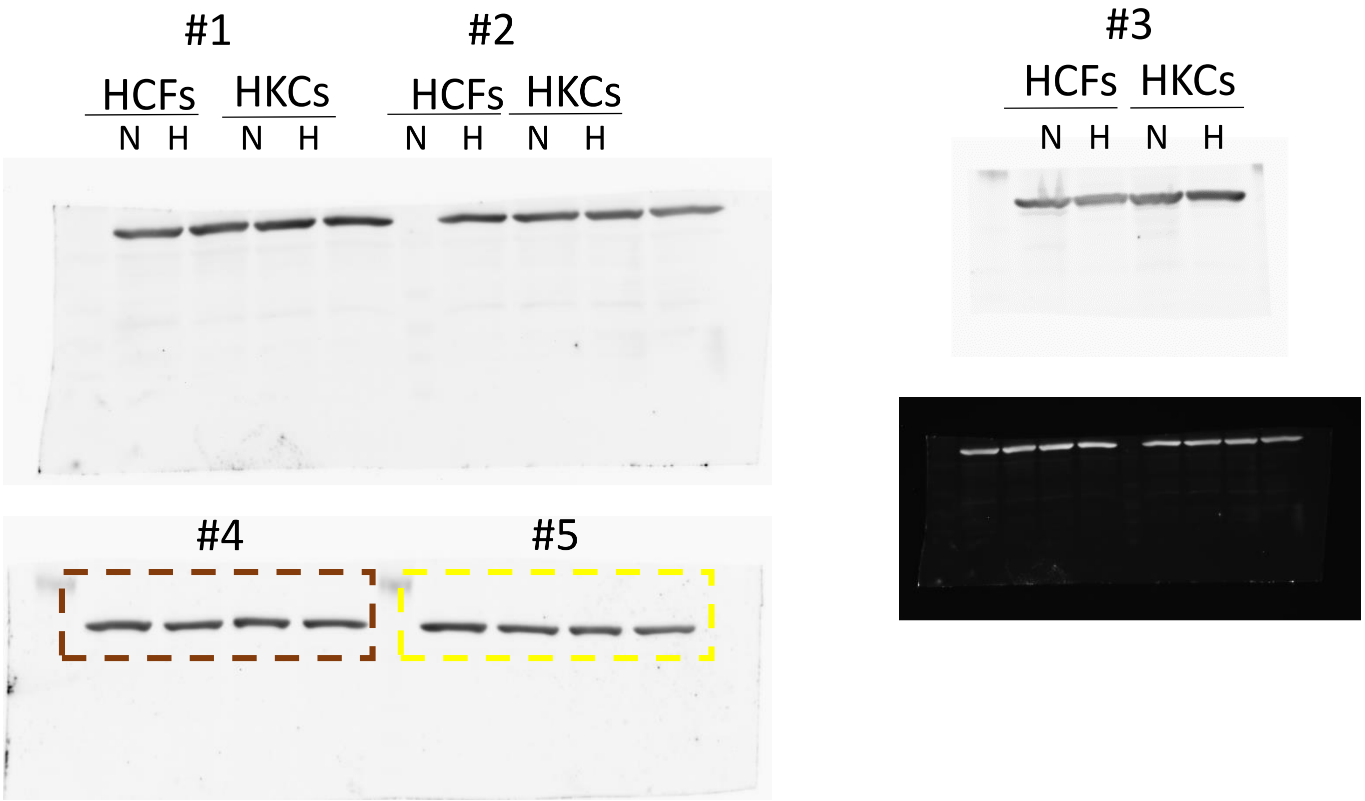

Uncropped, non-inverted blot

Uncropped blots of protein isolated from HCFs and HKCs following hypoxia exposure for 3 replicates. Dashed brown box denotes cropped area shown in Fig 3G, and dashed yellow box denotes cropped area shown in Fig 3L.

FAK

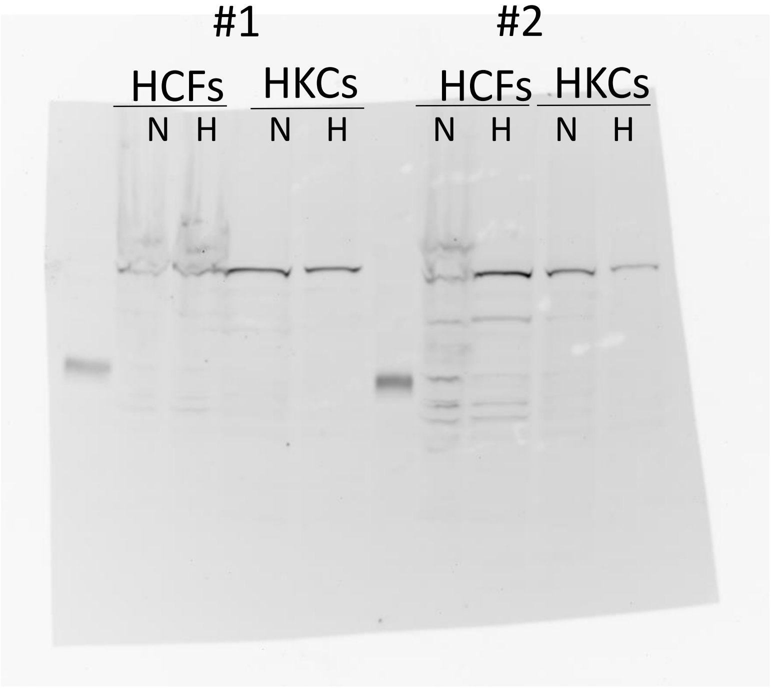

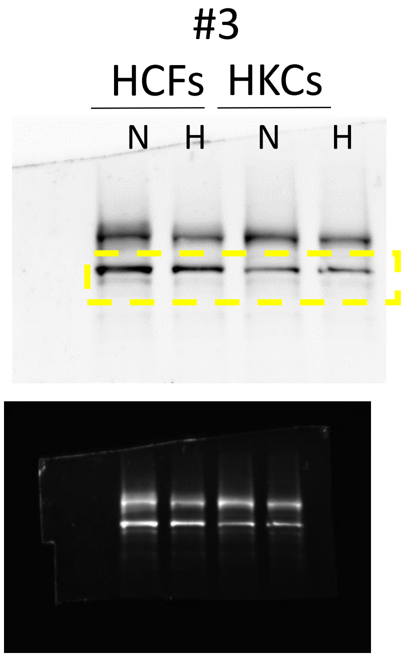

Uncropped, non-inverted blot

Uncropped blots of protein isolated from HCFs and HKCs following hypoxia exposure for 3 replicates. Dashed yellow box denotes cropped area shown in Fig 3L.

pFAK

#1

HCFs HKCs

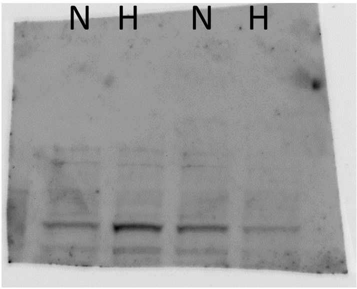

#2

HCFs HKCs

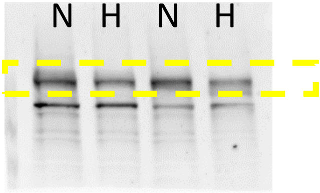

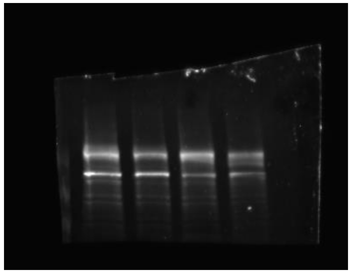

Uncropped, non-inverted blot

Uncropped blots of protein isolated from HCFs and HKCs following hypoxia exposure for 2 replicates. Dashed yellow box denotes cropped area shown in Fig 3L.
